# Epidemiological and imaging features that can affect the detection of ureterolithiasis on ultrasound

**DOI:** 10.1590/0100-3984.2017.0113

**Published:** 2018

**Authors:** Daniela Rebouças Nery, Yves Boher Costa, Thais Caldara Mussi, Ronaldo Hueb Baroni

**Affiliations:** 1 Hospital Universitário Professor Edgard Santos - Universidade Federal da Bahia (UFBA) e Delfin Medicina Diagnóstica, Salvador, BA, Brazil,; 2 MedRadius, Maceió, AL, Brazil,; 3 Hospital Israelita Albert Einstein, São Paulo, SP, Brazil.

**Keywords:** Ureterolithiasis, Ultrasonography, Tomography, X-ray computed, Body mass index, Ureterolitíase, Ultrassonografia, Tomografia computadorizada, Índice de massa corporal

## Abstract

**Objective:**

To identify, in patients with clinical suspicion of ureterolithiasis, epidemiological and imaging features that affect calculus detection on ultrasound, as well as to compare ultrasound with multidetector computed tomography (MDCT).

**Materials and Methods:**

We searched our database for patients who underwent ultrasound, followed by MDCT (if the ultrasound was negative), for suspected ureterolithiasis in an emergency setting. Patients were divided into three groups: positive ultrasound (US+); negative ultrasound/positive MDCT (US−/MDCT+); and negative ultrasound/negative MDCT (US−/MDCT−). We evaluated age, gender, ureterolithiasis laterality, location of the calculus within the ureter, body mass index, calculus diameter, and calculus attenuation on MDCT.

**Results:**

Of a total of 292 cases of suspected ureterolithiasis, 155 (53.1%) were in the US+ group, 46 (15.7%) were in the US−/MDCT+ group, and 91 (31.2%) were in the US−/MDCT− group. There were no significant differences among the groups in terms of age, gender, ureterolithiasis laterality, and mean MDCT attenuation values. Distal ureterolithiasis was most common in the US+ group, and calculi at other ureteral locations were more common in the US−/MDCT+ group. The mean body mass index was significantly higher in the US−/MDCT+ group than in the US+ group, and the mean calculus diameter was significantly greater in the US+ group than in the US−/MDCT+ group.

**Conclusion:**

A high body mass index, large calculus diameter, and calculus location in the distal third of the ureter are the major factors favoring ureterolithiasis detection on ultrasound.

## Abstract (pt)

**Objetivo:**

Identificar achados epidemiológicos e de imagem em pacientes com suspeita clínica de ureterolitíase que afetam a detecção do cálculo no ultrassom (US), comparado com a tomografia computadorizada multidetectores (TCMD).

**Materiais e Métodos:**

Procuramos, em nosso banco de dados, por pacientes que realizaram US no serviço de emergência, seguido por TCMD (se US negativo), por suspeita de ureterolitíase. Os pacientes foram divididos em: 1) US positivo; 2) US negativo e TCMD positiva; 3) US e TCMD negativos. Avaliamos idade, sexo, lateralidade, localização ureteral, índice de massa corporal, diâmetro e densidade do cálculo.

**Resultados:**

Foram incluídos no estudo 292 pacientes. Constatamos que 53,1% das ureterolitíases foram detectadas por US (grupo 1), 15,7% apenas por TCMD (grupo 2), e em 31,2% ambos os métodos foram negativos (grupo 3). Idade, sexo, lateralidade e densidade do cálculo pela TCMD não tiveram diferença significativa entre os grupos. No grupo 1, ureterolitíase foi detectada, preferencialmente, em localização distal, e fora do ureter distal no grupo 2. O índice de massa corporal foi significativamente maior em pacientes do grupo 2, comparado ao grupo 1. O diâmetro do cálculo detectado no grupo 1 foi significativamente maior do que no grupo 2.

**Conclusão:**

Índice de massa corporal, diâmetro e localização do cálculo no terço distal do ureter foram os principais fatores que contribuíram para a detecção do cálculo pelo US.

## INTRODUCTION

Ureterolithiasis causing acute flank pain is a common clinical situation in the emergency room^(1-3)^. Ultrasound is used extensively in the examination of patients with suspected ureterolithiasis and has the advantage of being universally available, fast, and easily performed, as well as not employing ionizing radiation^(1)^.

Ureterolithiasis can be accompanied by dilatation of the renal collecting system, depending on the size/location of the calculus, the duration of the resulting obstruction, and whether that obstruction is partial or complete^(4)^. Factors such as calculus size, patient weight, and body mass index (BMI) can influence ureterolithiasis detection by ultrasound^(5)^. In addition, the thickness of subcutaneous fat and its sound-attenuating properties reduce the accuracy of ultrasound^(6)^. Furthermore, the sensitivity of ultrasound depends on calculus size and location^(7)^. Fowler et al.^(8)^ found that ultrasound is a poor modality for identifying renal calculi smaller than 0.4 cm in diameter. Compared with computed tomography (CT), ultrasound has lower accuracy in the detection of ureterolithiasis, with a reported sensitivity and specificity of 11-93% and 95-100%, respectively^(5)^.

For a number of years, unenhanced multidetector CT (MDCT) has been the gold standard for diagnosing urinary tract calculi in adults^(3,9,10)^. The method reportedly has a sensitivity and specificity higher than 95% in detecting ureterolithiasis^(11,12)^. In addition, MDCT is suitable for detecting abnormalities that are unrelated to ureteral calculi. However, MDCT involves the use of radiation^(13)^, is more costly than ultrasound^(14)^, and, in rare cases, can present pitfalls in interpretation, such as mistaking a phlebolith for a distal ureterolithiasis^(15)^.

Given the higher cost of MDCT compared with ultrasound, together with the fact that a number of patients submitted to ultrasound will also need to undergo MDCT if the ultrasound findings are inconclusive^(16)^, one way to avoid diagnostic delays and to reduce costs would be to determine which patients would benefit from undergoing MDCT initially. It should also be borne in mind that, in most cases, ureterolithiasis is characterized by acute flank pain. Therefore, any attempt to expedite the diagnosis should be encouraged.

To our knowledge, there have been no studies investigating the effects that epidemiologic and topographical factors, as well as BMI, have on the detection of ureterolithiasis with ultrasound and MDCT. Therefore, the aim of the present study was to evaluate the epidemiological and imaging features that can affect the detection of ureterolithiasis on ultrasound.

## MATERIALS AND METHODS

### Study sample

This was a retrospective study of data related to the imaging examinations of patients who underwent ultrasound of the urinary tract in the emergency room, during a period of one year. We identified cases through searches of the database at our institution. We included cases in which the patient had positive ultrasound findings that explained the emergency room admission (ureterolithiasis or other condition requiring emergency treatment) or, if the ultrasound results were negative or inconclusive, had undergone MDCT within the first 24 h after the ultrasound. The exclusion criteria were as follows: having negative ultrasound findings and not having undergone MDCT to confirm the absence of disease; no epidemiological information being available; and images being unavailable. The local institutional review board approved the study and, because it was a retrospective study, waived the requirement for informed consent.

**Figure 1:**
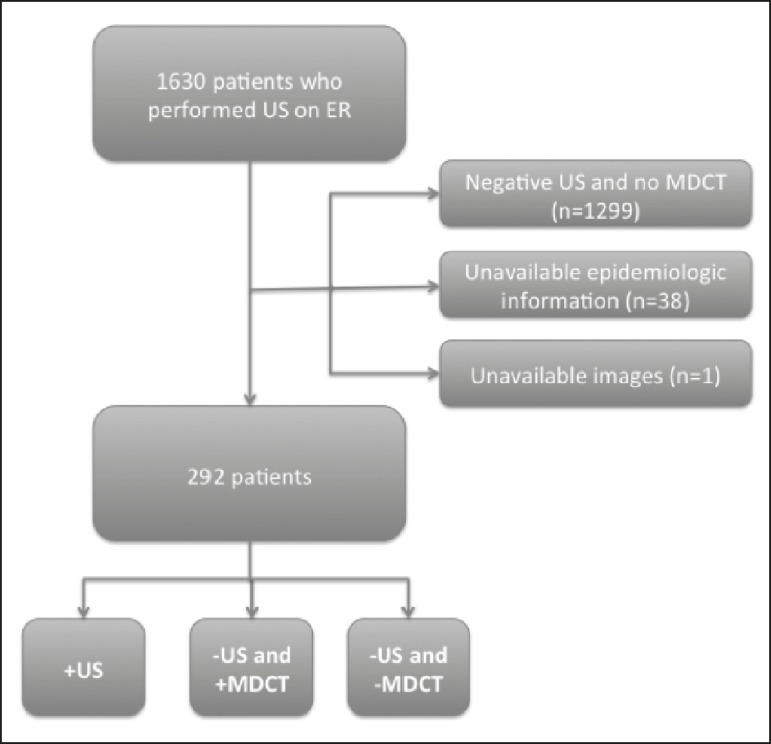
Flow chart of inclusion and exclusion criteria.

Of the 1630 patients who underwent ultrasound of the urinary tract during the study period, 1338 were excluded: because the ultrasound findings were negative and the patient did not undergo MDCT (n = 1299); because there was no epidemiological information available (n = 38); or because there were no images available (n = 1). Therefore, the final sample comprised 292 patients, who were divided into three groups (Figure 1): positive ultrasound (US+); negative ultrasound/positive MDCT (US−/MDCT+); and negative ultrasound/negative MDCT (US−/MDCT−).

**Figure 2:**
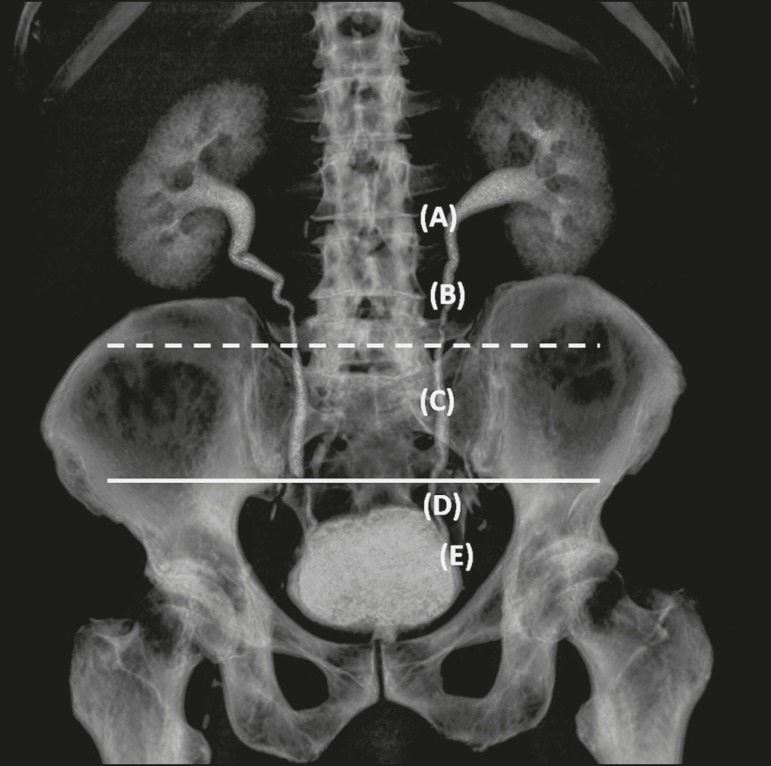
Three-dimensional reconstruction of an MDCT examination of the urinary tract. Note the ureteral anatomic division: the UPJ (A); the proximal ureter (B); the mid-ureter (C), between the upper border of sacroiliac joint (dashed line) and its lower border (solid line); the distal ureter (D); and the UVJ (E).

Epidemiological and topographical factors were compared among the groups. The parameters analyzed were the age, gender, and BMI of the patient, together with the laterality, size, location, and attenuation of the ureterolithiasis. The location of the calculus in the ureter was classified as follows (Figure 2): the ureteropelvic junction (UPJ); the proximal ureter (portion of the ureter that extends from the UPJ to the upper border of the sacroiliac joint); the mid-ureter (between the upper and lower borders of the sacroiliac joint); the distal ureter (portion of the ureter that extends from lower border of the sacroiliac joint to the ureterovesicular junction [UVJ]); and the UVJ. For the patients who had undergone both types of examinations, MDCT was considered the reference.

### Imaging

Ultrasound examinations were performed with one of two different ultrasound systems (HDI 5000 or IU22; Philips Medical Systems, Amsterdam, the Netherlands). Curved phased-array (3.5-5.0 MHz) transducers were used. All examinations were conducted by radiologists with at least four years of experience in evaluating abdominal ultrasounds. Ureteral calculi were measured at their longest diameter.

MDCT examinations were performed in 64- or 320-detector row scanners (Aquilion 64 or Aquilion One; Toshiba Medical Systems, Tokyo, Japan). In accordance with the protocol of our institution, there was no oral or intravenous administration of contrast medium. MDCT images extended from the upper poles of the kidneys through the pubic symphysis. A board-certified radiologist, blinded to the ultrasound results, reviewed the MDCT examinations, measured the calculus size (on its longitudinal and perpendicular axis), and determined its attenuation (in HU).

### Statistical analysis

Statistical analyses were performed with the Statistical Package for the Social Sciences, version 17.0 (SPSS Inc., Chicago, IL, USA) and with the program R (R Development Core Team, 2012). Univariate analysis was performed with chi-square or Fisher's exact tests to analyze each categorical variable (gender, ureterolithiasis laterality, and calculus location). One-way analysis of variance was performed to analyze continuous variables (age, BMI, calculus size, and calculus density). In a multivariate analysis, we performed stepwise logistic regression to identify factors that predicted the detection of ureterolithiasis by ultrasound. The analyses were performed with the Statistical Analysis System software, version 9.3 (SAS Institute Inc., Cary, NC, USA). Receiver operating characteristic (ROC) curve analysis was performed with the Number Cruncher Statistical System program, version 2004 (NCSS, Kaysville, UT, USA). For all analyses, values of *p* < 0.05 were considered statistically significant.

## RESULTS

Ultrasound was performed in all 292 patients in the sample (168 men, 124 women; mean age, 47.7 years). Of those 292 patients, 155 (53.1%) were in the US+ group, 46 (15.7%) were in the US−/MDCT+ group, and 91 (31.2%) were in the US−/MDCT− group.

### Ureterolithiasis laterality

Of the 155 calculi identified by ultrasound, 85 (54.8%) were in the left ureter and 70 (45.2%) were in the right ureter. Of the 46 calculi identified only by MDCT, 26 (56.5%) were in the left ureter and 20 (43.5%) in the right ureter. The laterality of the calculi did not differ significantly different between the US+ and US−/MDCT+ groups (*p* = 0.954).

### Calculus location

Of the 155 calculi identified by ultrasound, 12 (7.8%) were in the UPJ, 17 (11.0%) were in the proximal ureter, 14 (9.1%) were in the mid-ureter, 63 (40.9%) were in the distal ureter, and 48 (31.2%) were in the UVJ. Of the 46 calculi identified only by MDCT, 1 (2.2%) was in the UPJ, 10 (21.7%) were in the proximal ureter, 10 (21.7%) were in the mid-ureter, 16 (34.7%) were in the distal ureter, and 9 (19.5%) were in the UVJ. The proportion of patients with calculi in distal locations (the distal ureter or UVJ) was highest in the US+ group (*p* = 0.036).

### BMI

**Figure 3:**
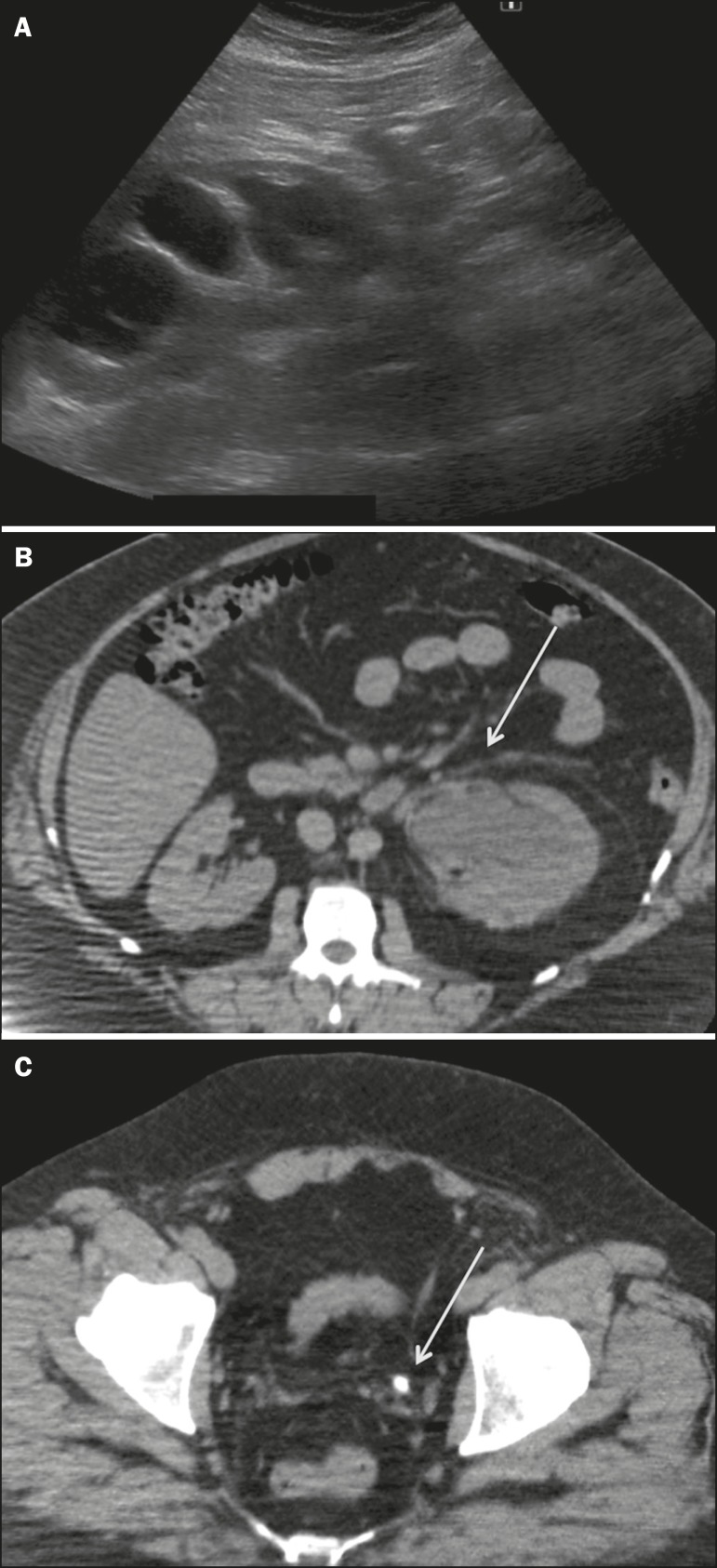
A 43-year-old male, BMI = 39, with abdominal pain. Ultrasound image (**A**) showing marked left collecting system dilatation without detecting the obstructive cause. Following ultrasound, MDCT was performed which confirmed the collecting system dilatation (**B**) and a calculus in the distal ureter (**C**) (arrows), measuring 0.6 cm.

The mean BMI was 25 kg/m^2^ in the US+ group, 27 kg/m^2^ in the US−/MDCT+ group, and 24 kg/m^2^ in the US−/MDCT− group, the difference between the US−/MDCT+ group and the two other groups being statistically significant (*p* = 0.028). Figure 3 illustrates the case of a patient with a BMI of 39 kg/m^2^, in whom ultrasound failed to detect the ureterolithiasis, which was diagnosed on the basis of the MDCT findings (US−/MDCT+ group).

### Calculus diameter and MDCT attenuation

When determined by ultrasound, the mean diameter of the calculi was 0.62 ± 0.3 cm (range, 0.1-1.6 cm), compared with 0.46 ± 0.2 cm (range, 0.2-1.0 cm) when it was determined by MDCT (in the transverse plane). The mean diameter of the calculi was greater in the US+ group than in the US−/MDCT+ group (0.62 cm vs. 0.46 cm), and the difference was significant (*p* < 0.001). The mean MDCT attenuation for the calculi was slightly higher in the US+/MDCT+ group than in the US−/MDCT+ group (1117 ± 349 HU vs. 902 ± 394 HU), although the difference was not statistically significant (*p* = 0.235).

### Epidemiological factors

There were no statistical differences among the groups in terms of the mean age (*p* = 0.821) or the gender distribution (*p* = 0.589).

### Differential diagnoses of acute abdominal pain

Acute abdominal pain was attributed to a cause other than ureterolithiasis in 33 (11.3%) of the 292 patients. Of those 33 patients, 20 (60.6%) were female and 13 (39.4%) were male. Two of those 33 patients were diagnosed with hemorrhagic ovarian cyst; six patients were diagnosed with pyelonephritis; 14 patients were diagnosed with pyeloureteritis cystica; and one patient each was diagnosed with acute prostatitis, diverticulitis, omental infarction, focal pyelonephritis, bladder calculi, nephrocalcinosis, polycystic kidney with bleeding, lumbar vertebral fracture, ureteral stenosis, and urothelial tumor.

### Multivariate analyses and ultrasound false-negative results

**Figure 4:**
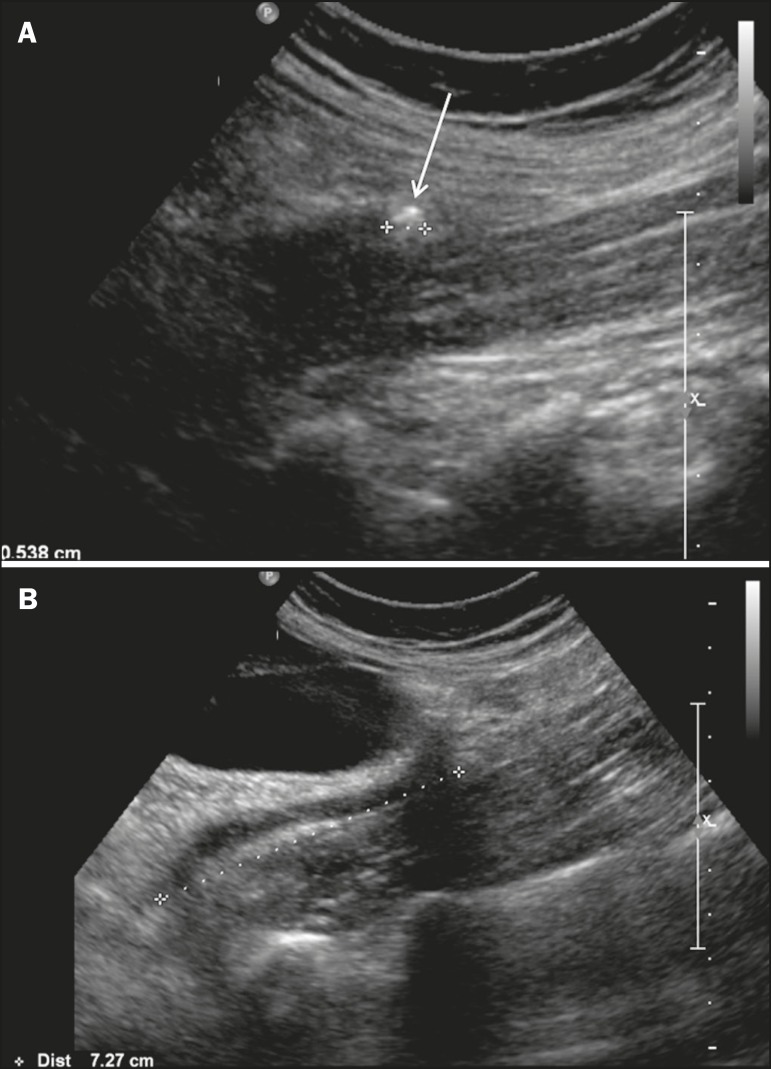
A 47-year-old female with a BMI of 22 kg/m2 and ureteral colic. Ultrasound showing a calculus measuring 0.5 cm (arrow) in the mid-ureter (**A**), 7.0 cm below the renal pelvis (**B**).

Using logistic regression (Table 1), we found that a BMI ≤ 27 kg/m^2^, a calculus diameter (in the transverse plane on MDCT) of 0.5-0.7 cm, and location of the calculus in the distal ureter were the best predictors of ultrasound detection, with respective correlation coefficients of 1.15 (vs. > 27 kg/m^2^), 0.61 (vs. < 0.5 cm), and 0.08 (vs. a proximal location). Figure 4 illustrates the case of a patient in which a calculus with a diameter of 0.5 cm, located in the mid-ureter, was detected by ultrasound (US+ group).

**Table 1:** Multivariate logistic regression analysis of factors potentially predictive of false-negative ultrasound results for ureterolithiasis (n = 201).

Independent variables	*P*-value	OR (95% CI)
Age (each year)	0.821	1 (0.97-1.04)
BMI (each unit)	0.007*	5 (1.04-1.28)
Ureterolithiasis laterality (left vs. right)	0.785	1.12 (0.5-2.48)
Gender (female vs. male)	0.589	1.28 (0.53-3.08)
Calculus diameter		
(< 0.5 cm vs. 0.5-0.7 cm)	< 0.001*	3.54 (1.53-8.21)
(> 0.7 cm vs. 0.5-0.7 cm)	0.002*	0.19 (0.04-0.8)
Calculus location		
(UPJ vs. distal)	0.663	1.04 (0.11-10.24)
(Proximal vs. distal)	0.018*	4.56 (1.47-14.18)
(Mid-ureter vs. distal)	0.058	3.75 (1.24-11.34)
(UVJ vs. distal)	0.011*	0.5 (0.19-1.35)

OR, odds ratio; CI, confidence interval.

For each unit increase in BMI, we observed an increase of 16% in likelihood of false-negative ultrasound results. In addition, the likelihood of a false-negative ultrasound result increased 234% for calculi with a diameter < 0.5 cm than for those with a diameter of 0.5-0.7 cm, whereas increased 426% if the calculus was located in the proximal ureter than if it was located in the distal ureter. For each millimeter increase in the size of the calculus, the likelihood of false negative of ureterolithiasis by ultrasound decreased 40% (*p* < 0.001).

### Determination of the optimal BMI cut-off value for ultrasound detection of ureterolithiasis

**Figure 5:**
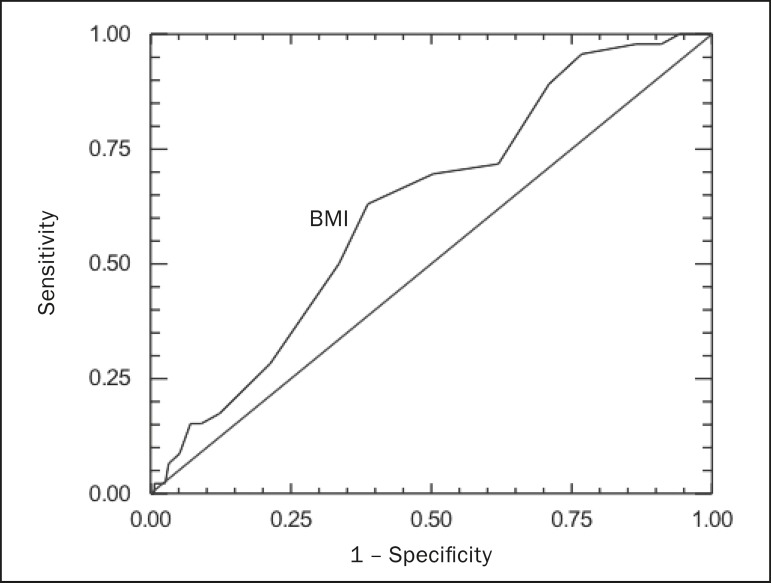
ROC curve analysis for ultrasound detection of ureterolithiasis, according to BMI.

Using a ROC curve analysis (Figure 5), we found that, for optimal detection of ureterolithiasis by ultrasound, patients should have a BMI ≤ 27 kg/m^2^ (*p* = 0.005). That cut-off value was found to have a sensitivity, specificity, positive predictive value, and negative predictive value of 63.0%, 61.3%, 33.0% and 95.0%, respectively, for the ultrasound detection of ureterolithiasis.

## DISCUSSION

Choosing the correct initial imaging modality is an essential part of the management of cases in which the patient presents to the emergency department with flank pain. Various imaging modalities are available to evaluate hydronephrosis and renal calculi (conventional X-ray of the abdomen; specific X-ray examination of the kidney, ureter, or bladder; ultrasound; MDCT; and magnetic resonance imaging), although most recent protocols limit the choices of initial imaging modalities in an acute setting to MDCT and ultrasound^(17,18)^.

In the present study, we aimed to determine which epidemiological and imaging findings should be considered when selecting the first-line diagnostic tool for the identification of ureterolithiasis. In addition, we attempted to determine to what degree the size and location of a calculus in the ureter can affect the ability of ultrasound to detect it.

According to our findings, the use of ultrasound in patients with a BMI > 27 kg/m^2^ can delay diagnosis and treatment because a greater number of patients will also need to undergo MDCT to detect the calculi. One previous report showed that protocols involving early CT can reduce the overall cost by shortening hospital stays^(19)^.

Previous studies have reported that MDCT has high sensitivity and specificity (97% and 96%, respectively) for the detection of ureterolithiasis^(11,12)^. For ultrasound, the reported sensitivity for the detection of ureterolithiasis varies widely, from 11% to 93%^(5,7,14,20)^. In addition, ultrasound can overestimate the calculus size, especially for calculi with a diameter ≤ 0.5 cm^(5)^. The accurate measurement of the diameter of a calculus and the determination of its location are essential to predicting the chances of its spontaneous passage and defining its correct management^(21)^.

Previous studies have considered a cut-off BMI of > 30 kg/m^2^ to choose between low-dose and standard CT protocols^(17,22)^. However, to our knowledge, there have been no studies taking patient BMI into consideration when evaluating the efficacy of ultrasound for ureterolithiasis detection. According to our results, ultrasound is of limited value for diagnosing ureterolithiasis in patients with a BMI > 27 kg/m^2^. In fact, in an obese patient with 8 cm of subcutaneous fat, 94% of the ultrasound wave is absorbed before it reaches the peritoneal cavity^(23)^. The effect of obesity on the accuracy of ultrasound in detecting ureterolithiasis has not been investigated in depth. Ulusan et al.^(6)^ found no correlation between BMI and the rate of detection of renal calculi with ultrasound, although their study involved only renal calculi and not ureteral calculi.

We found that most of the ureteral calculi identified on ultrasound were located in the distal third of the ureter, especially in the UVJ, which is similar to the findings of other studies^(1,13)^. Distal locations (the distal ureter and UVJ) accounted for 72.1% of all such calculi. Other studies have also shown that the detection ability of ultrasound varies depending on the location of the calculus in the ureter^(1,13)^.

Ureteral calculi < 0.5 cm in diameter can be overlooked on ultrasound because they do not cast an acoustic shadow and may not be distinguishable from the normally hyperechoic renal sinus^(6)^. Previous research has demonstrated that the sensitivity of ultrasound for renal calculus detection is dependent on calculus size^(8)^. In the present study, we found significantly different detection rates for the different sizes of calculi.

Some epidemiological factors, such as gender, can be important to define the presence of ureterolithiasis. In the present study, the incidence of back pain was higher among premenopausal women than among men, although all such women were in the US−/MDCT− group (i.e., no ureterolithiasis was detected) and the difference was not statistically significant. That finding might be related to a discretely higher incidence of pyelonephritis among the females in the sample.

Ulusan et al.^(6)^ reported that, using ultrasound, it was more difficult to visualize small renal calculi in the left kidney than in the right kidney. However, we observed no significant difference between the right and left ureters in terms of the sensitivity of ultrasound for the detection of calculi.

Of the 292 patients in our sample, 33 (11.3%) presented a diagnosis other than ureteral calculus. Similar incidences of alternative or additional causes to flank pain have previously been observed^(24,25)^, which supports the recommendation for additional investigation after an initial imaging examination excludes ureterolithiasis.

Our study has some limitations. It was a retrospective study of data obtained with different types of MDCT scanners and ultrasound machines, which might have introduced some variability into our results. In addition, a large number of patients were excluded because they had negative ultrasound results and did not undergo MDCT within the following 24 h. Furthermore, a number of radiologists with different levels of experience analyzed the ultrasound images. However, at many facilities, ultrasound is performed by a radiology technician rather than a radiologist. Finally, we had no access to patient histories, which could be a limitation, because some patients could have had prior episodes of ureterolithiasis.

We conclude that, among the patient-related variables that can be measured prior to imaging, BMI is the only one that can be taken into consideration when trying to decide whether ultrasound or MDCT should be performed first. Because there is no way of knowing the location or size of the ureteral calculus beforehand, the findings that calculus size and location in the ureter affect ultrasound detection of ureterolithiasis have no clinical relevance.
